# Prevalence of antiphospholipid autoantibodies associated with biologics treatment for psoriasis

**DOI:** 10.1038/s41598-024-65378-6

**Published:** 2024-07-10

**Authors:** Lixin Li, Satoshi Toyama, Yuka Mizuno, Toyoki Yamamoto, Asahi Hiroshima, Asumi Koyama, Haruka Taira, Eiki Sugimoto, Yukiko Ito, Kentaro Awaji, Shoko Tateishi, Hiroko Kanda, Yoshihide Asano, Shinichi Sato, Sayaka Shibata

**Affiliations:** 1 Department of Dermatology, Graduate School of Medicine, The University of Tokyo, 7-3-1 Hongo, Bunkyo-Ku, Tokyo, 113-8655 Japan; 2 Department of Allergy and Rheumatology, Graduate School of Medicine, The University of Tokyo, Tokyo, Japan; 3 Division for Health Service Promotion, University of Tokyo, Tokyo, Japan; 4 Immune-Mediated Diseases Therapy Center, Graduate School of Medicine, University of Tokyo, Tokyo, Japan; 5 Department of Dermatology, Tohoku University Graduate School of Medicine, Sendai, Japan

**Keywords:** ANA, APS, Autoantibodies, Psoriasis, Biologics, Skin diseases, Outcomes research

## Abstract

Psoriasis is a chronic inflammatory disease that sometimes necessitates therapeutic intervention with biologics. Autoantibody production during treatment with tumor necrosis factor (TNF) inhibitors is a recognized phenomenon, however, the production of autoantibodies associated with antiphospholipid syndrome (APS) has not been comprehensively evaluated in patients with psoriasis. This study was conducted to assess the prevalence of APS-associated autoantibodies in patients with psoriasis treated with different biologics and to investigate the potential associations between autoantibody production and clinical or serological parameters. Patients with psoriasis undergoing biologics treatments were enrolled in this study, and were categorized based on the type of biologics administered, TNF, interleukin (IL)-17, or IL-23 inhibitors. Clinical and serological data were collected and analyzed in conjunction with data on APS autoantibodies. TNF inhibitors were associated with a higher frequency of APS autoantibodies compared to IL-17 and IL-23 inhibitors. Notably, the presence of APS autoantibodies correlated with concurrent arthritis and higher disease severity at treatment initiation in patients treated with TNF inhibitors. Elevated Psoriasis Area and Severity Index scores and anti-nuclear antibody titers higher than × 320 were predictors of APS autoantibody production. Despite the higher autoantibody rates, clinical symptoms of APS were absent in these patients. This study provides the first comprehensive evidence of an increased frequency of APS autoantibodies associated with TNF inhibitor treatment in patients with psoriasis. The observed association between APS autoantibody positivity and TNF inhibitor treatment or clinical parameters suggests a potential immunomodulatory interplay between autoimmunity and inflammation in the pathogenesis of psoriasis.

## Introduction

Psoriasis is a chronic inflammatory disorder that involves both innate and adaptive immune systems in the development and maintenance of the disease^1–3^. Biologics that specifically inhibit the target factors, including tumor necrosis factor (TNF), interleukin (IL)-23, and IL-17, have been used worldwide with well-established clinical efficacy^4^. While biologics have a low incidence of adverse effects such as hepatic or renal dysfunction compared to small molecule drugs, their administration, particularly in the case of TNF inhibitors (TNFi), is sometimes associated with the development of autoantibodies^5^. The most frequently observed autoantibodies are antinuclear antibodies (ANA), and their occurrence has been noted in up to approximately 70% of patients receiving TNFi^5–7^.

The presence of these autoantibodies may not necessarily have pathological significance, however, as a rare adverse event, they cause a clinically evident autoimmune condition, including lupus symptoms, vasculitis, and demyelinating disorders^5,8^. We previously reported a case of chronic inflammatory demyelinating polyneuropathy that manifested during the treatment of adalimumab with the development of an anti-ganglioside GM1 IgM antibody and improved upon discontinuation in a patient with psoriasis vulgaris^8^. The incidence of autoantibodies depends on the underlying disease conditions and the biologics being used. Among autoantibodies, the development of autoantibodies associated with antiphospholipid syndrome (APS), those targeting antiphospholipid antigens, autoantibodies directed against cardiolipin (aCL-IgG), and β2-glycoprotein I (anti-β2GPI), and lupus anticoagulants (LAC), have been reported in patients with rheumatoid arthritis treated with TNFi^9,10^. However, the correlations between these autoantibodies and the types of biologics administered remain inconclusive in psoriasis.

In this study, a retrospective analysis was conducted to investigate the incidence of ANA and APS-associated autoantibodies in psoriasis patients following treatment with TNF, IL-17, and IL-23 inhibitors. Furthermore, the present study investigated the factors predisposing patients to developing APS autoantibodies.

## Methods

### Patients

A retrospective analysis was performed on patients diagnosed with psoriasis vulgaris, psoriatic arthritis, or generalized pustular psoriasis by dermatologists and treated with biologics from January 2010 to December 2021 at the University of Tokyo Hospital (Tokyo, Japan). Patients with palmoplantar pustulosis were not included in the present study. In this retrospective study, an opt-out consent method was approved and utilized, as granted by the medical ethics committee of the University of Tokyo (No. 3360). Participants were provided with comprehensive information about the study and were given the opportunity to decline participation. All procedures were carried out in accordance with ethical standards of the responsible committee on human experimentation and with the Helsinki Declaration of 1975, as revised in 2013.

### Clinical assessments and data collection

Baseline information included age, sex, Body Mass Index (BMI), and disease duration before the initiation of biologics. The presence of clinical comorbidities, including arthritis, diabetes, hypertension, and dyslipidemia was also investigated. Disease severity was assessed by a dermatologist using Psoriasis Area Severity Index (PASI) scores^11^, and arthritis was diagnosed by rheumatologists based on the Classification Criteria for Psoriatic Arthritis (CASPAR) criteria^12^. The hematological laboratory data of patients were extracted from our registry.

### Statistical analysis

Kruskal–Wallis test with Dunn–Bonferroni post hoc test for multiple comparisons and Mann–Whitney’s U-test for two-group comparisons were conducted for continuous variables. Fisher’s exact test with Bonferroni post hoc test was used for group comparisons of categorical variables. Multivariate Cox regression analyses were conducted to examine the association between predictive variables and the probability of positive APS autoantibodies. *P* < 0.05 was considered statistically significant throughout all the analyses. Statistical analyses were performed using the Prism 8 and 9 (Graph Pad Software, CA, USA) and EZR software program^13^.

## Results

### Frequency of ANA-positive conversion and APS autoantibody positivity stratified by the type of biologics

This retrospective cohort study included 286 patients with psoriasis undergoing biologics treatment. Patients were categorized into three groups based on the type of biologics used in their treatment: TNFi, IL-17 inhibitors (IL-17i), and IL-23 inhibitors (IL-23i) (Table 1). In each treatment group, baseline ANA positivity rates did not differ significantly, with rates of 20–30% for a cutoff value of 1:40 and up to 5% for a cutoff of 1:320, which are generally consistent with the rates on healthy controls available from our facility. The proportion of patients whose ANA status converted to positive after administration of each treatment was then examined (Table 2). Some cases in the IL-17i and IL-23i groups were later switched to TNFi treatment; these cases were excluded from further analyses. Since the threshold titer of ANA depends on reagents, instruments, and local factors, two patterns of thresholds, one with a threshold of 40 × and another with a higher threshold of 320x, were set for positive outcomes in the present study. For both threshold settings, the rates of ANA-positive conversion were higher in TNFi-treated patients compared to IL-17i or IL-23i-treated patients (*P* < 0.001). Of the 30 patients with post-treatment titers ≥ 320x, 19 were initially pre-treatment ANA negative, while the remaining 11 had baseline titers of 40 × or 160x. Excluding these 11 patients, the percentage of positive conversion from ANA negative to titers ≥ 320 × was still significantly higher in the TNFi-treated group (19 out of 122) compared to the IL-17i (none out of 31) or IL-23i (none out of 26)-treated group (*P* = 0. 042 vs. IL-17i and *P* = 0. 077 vs. IL-23i by Fisher’s exact test with Bonferroni post hoc test). The duration from the initiation of biologics to ANA-positive conversion showed no statistical difference between the therapeutic agents, indicating that the timing of positive conversion may be more influenced by individual differences than by the drug type. The frequency of post-treatment APS-associated autoantibodies, aCL-IgG, anti-β2GPI, and LAC in each group was also examined. There was a statistically significant difference in the overall frequency of these autoantibodies among the three treatment groups (*P* = 0.01), with TNFi-treated patients showing a higher prevalence. However, this difference did not reach significance when analyzing the prevalence of individual antibodies separately. Notably, none of the patients who were positive for APS autoantibodies exhibited clinical symptoms of thrombosis or embolism. Five patients with relatively high antibody titers underwent intravascular echography to assess for potential thrombosis, yet the examination revealed no significant findings.

**Table 1:** Baseline clinical characteristics of psoriasis patients categorized by treatment group.

	TNFi	IL-17i	IL-23i	*P* value
Number of patients	170	60	56	
Sex				
M/F, No	127/43	33/27	38/18	.02^a^
Initiation age of biologics, years (median, IQR)	53.0 (45.0–62.0)	56.0 (49.0–69.0)	53.5 (37.0–66.0)	.11
BMI (median, IQR)	25.2 (22.1–28.1)	25.4 (22.6–28.0)	24.0 (21.9–26.5)	.39
Type of psoriasis				
PsA, No	70	27	12	< .01^b^
PsV, No	85	33	44	
GPP, No	15	0	0	
PASI (median, IQR)	6.6 (3.8–16.2)	7.1 (4.5–14.8)	7.5 (4.5–17.7)	.96
Concurrent HT or DL or DM, No. (%)	98/140 (70.0%)	31/46 (67.4%)	24/49 (49.0%)	.03^c^
Disease duration prior to the initiation of biologics, years (median, IQR)	14.0 (6.0–24.0)	14.0 (8.0–24.0)	10.0 (4.0–21.8)	.55
ANA positivity (titers higher than 1:40)	36/158 (22.8%)	15/49 (30.6%)	9/40 (22.5%)	.51
ANA positivity (titers higher than 1:320)	1/158 (0.6%)	0/49 (0.0%)	2/40 (5.0%)	.10

^a^*P* value with 0.02 between TNFi and IL-17i group by Bonferroni test as post hoc comparison.

^b^*P* value < .001 between TNFi and IL-23i group and *P* value with 0.03 between IL-17i and IL-23i group by Bonferroni test as post hoc comparison.

^c^*P* value with 0.03 between TNFi and IL-23i group by Bonferroni test as post hoc comparison.

*TNFi* tumor necrosis factor inhibitor, *IL-17i* interleukin-17 inhibitor, *IL-23i* interleukin-23 inhibitor, *M* male, *F* female, *IQR* interquartile range, *PsA* psoriatic arthritis, *PsV* psoriasis vulgaris, *GPP* generalized pustular psoriasis, *PASI* Psoriasis Area and Severity Index, *HT* hypertension, *DL* dyslipidemia, *DM* diabetes mellitus.

**Table 2:** Post-treatment rates of ANA-positive conversion and APS autoantibody positivity in psoriasis patients categorized by treatment group.

	TNFi (n = 170)	IL-17i (n = 60)	IL-23i (n = 56)	*P* value
ANA-positive conversion rates (titers higher than 1:40)	82/122 (67.2%)	8/31 (25.8%)	9/26 (34.6%)	< .001^a^
ANA pattern No. (%)				
Centromere type	2/82 (2.4%)	0/8 (0.0%)	0/9 (0.0%)	1
Homogeneous type	63/82 (76.8%)	4/8 (50.0%)	4/9 (4.4%)	.04
Nucleolar type	25/82 (30.5%)	2/8 (25.0%)	3/9 (33.3%)	1
Speckled type	55/82 (67.1%)	7/8 (87.5%)	7/9 (77.8%)	.57
ANA-positive conversion rates (titers higher than 1:320)	30/157 (19.1%)	0/39 (0.0%)	1/34 (2.9%)	< .001 ^b^
ANA pattern No. (%)				
Centromere type	1/30 (3.3%)	NA	0/1 (0.0%)	1
Homogeneous type	27/30 (90.0%)	NA	0/1 (0.0%)	.13
Nucleolar type	5/30 (16.7%)	NA	0/1 (0.0%)	1
Speckled type	5/30 (16.7%)	NA	0/1 (0.0%)	1
Duration from the initiation of biologics to ANA-positive conversion (titers higher than 1:40), months (median, IQR)	11.0 (5.0–29.8)	14.5 (4.0–34.8)	6.0 (1.0–24.0)	.63
Duration from the initiation of biologics to ANA-positive conversion (titers higher than 1:320), months (median, IQR)	19.0 (8.0–35.3)	NA	23.0 (12.0–26.5)	.22
APS autoantibody positivity, No. (%)	54/113 (47.8%)	14/37 (37.8%)	5/28 (17.9%)	.01^c^
LAC positivity, No. (%)	41/113 (36.3%)	12/37 (32.4%)	4/28 (14.3%)	.07
aCL-IgG positivity, No. (%)	19/108 (17.6%)	2/32 (6.3%)	1/26 (3.8%)	.10
aβ2GPI positivity, No. (%)	4/108 (3.7%)	0/33 (0.0%)	0/26 (0.0%)	.78

^a^*P* value < .001 between TNFi and IL-17i and *P* value with 0.01 between TNFi and IL-23i group by Bonferroni test as post hoc comparison.

^b^*P* value < .005 between TNFi and IL-17i and *P* value with 0.058 between TNFi and IL-23i group by Bonferroni test as post hoc comparison.

^c^*P* value with 0.015 between TNFi and IL-23i group by Bonferroni test as post hoc comparison.

*ANA* antinuclear antibody, *APS* antiphospholipid syndrome, *LAC* lupus anticoagulant, *aCL-IgG* anticardiolipin antibodies IgG, *aβ2GPI* anti-beta2-glycoprotein I antibodies.

### Clinical features of patients exhibiting ANA-positive conversion and APS autoantibody positivity

Given the higher prevalence of ANA-positive conversion and APS autoantibodies in psoriasis patients treated with TNFi, subsequent analyses focused on a subset of TNFi-treated patients. Initially, a comparison was conducted between patients who developed ANA positivity and those who did not, focusing on clinical symptoms and laboratory results (Table 3). No statistically significant differences were observed concerning gender, age, PASI scores, disease duration, and prevalence of hypertension, dyslipidemia, or diabetes mellitus. Notably, patients with ANA-positive conversion exhibited a significantly higher prevalence of concurrent arthritis (*P* = 0.04) and lower BMI (*P* = 0.03) compared to their counterparts. Clinical symptoms were also compared between patients with positive and negative for APS autoantibodies (Table 4). Similarly, APS autoantibody-positive patients showed a higher prevalence of concurrent arthritis than their APS autoantibody-negative counterparts (*P* = 0.03). Furthermore, APS autoantibody-positive patients had higher PASI scores compared to APS autoantibody-negative patients (*P* = 0.02). Given the reported association between the emergence of ANA and loss of treatment response to TNFi^7,14^, the frequency of biologics switches was examined to assess treatment responsiveness in APS autoantibody-positive patients. Our analysis revealed no significant difference in the number of therapeutic switches between APS autoantibody-positive and negative patients.

**Table 3:** Clinical characteristics of psoriasis patients treated with TNFi, categorized by ANA-positive conversion.

	ANA-positive conversion		
	Positive (n = 82)	Negative (n = 40)	*P* value
Sex			
M/F	61/21	32/8	.65
Initiation age of biologics, years (median, IQR)	52.0 (45.3–57.8)	53.0 (39.0–62.0)	.78
BMI (median, IQR)	24.9 (21.2–27.6)	27.1 (22.9–30.5)	.03
PASI (median, IQR)	6.3	5.6	.33
	(3.6–15.8)	(3.0–10.8)	
Concurrent HT or DL or DM, No. (%)	48/67 (71.6%)	22/33 (66.7%)	.65
Disease duration prior to the initiation of biologics, years (median, IQR)	18.0	14.0	.23
	(7.0–27.0)	(6.0–20.0)	
Concurrent arthritis at the initiation of biologics, No. (%)	32/82 (39.0%)	8/40 (20.0%)	.04

*ANA* antinuclear antibody, *M* male, *F* female, *IQR* interquartile range, *BMI* body mass index, *PASI* Psoriasis Area and Severity Index, *HT* hypertension, *DL* dyslipidemia, *DM* diabetes mellitus.

**Table 4:** Clinical characteristics of psoriasis patients treated with– TNFi, categorized by positivity for APS autoantibody.

	APS autoantibody		
	Positive (n = 54)	Negative (n = 59)	*P* value
Sex			
M/F	41/13	42/17	.67
LAC (median, IQR)	1.4 (1.3–1.5)	1.2 (1.1–1.2)	< .001
aCL-IgG (median, IQR)	8.0 (4.0–54.0)	4.3 (4.0–7.8)	< .01
aβ2GPI (mean, IQR)	1.8 (1.3–1.3)	1.3 (1.3–1.3)	< .01
Initiation age of biologics, years (median, IQR)	51.0	53.0	.45
	(45.0–61.0)	(45.0–60.0)	
BMI (median, IQR)	25.1	25.2	.29
	(22.9–28.5)	(21.8–27.2)	
PASI (median, IQR)	11.9	6.3	.02
	(5.5–23.7)	(3.9–14.7)	
Concurrent HT or DL or DM, No. (%)	38/46 (82.6%)	32/49 (65.3%)	.07
Disease duration prior to the initiation of biologics, years (median, IQR)	13.0	15.5	.39
	(6.0–25.0)	(7.3–25.8)	
Concurrent arthritis at the initiation of biologics, No. (%)	27/54 (50.0%)	17/59 (28.8%)	.03
Biological switch times			.68
0	21	17	
1	19	25	
2	8	8	
3 times or more	6	9	

*APS* antiphospholipid syndrome, *M* male, *F* female, *IQR* interquartile range, *BMI* body mass index, *PASI* Psoriasis Area and Severity Index, *HT* hypertension, *DL* dyslipidemia, *DM* diabetes mellitus, *ANA* antinuclear antibody.

### Prediction of developing APS autoantibodies

Multivariate analyses were performed to explore the potential predictive correlations of various clinical characteristics and laboratory data with the subsequent development of APS autoantibodies (Table 5). Higher PASI scores at the initiation of biologics (HR 1.11, *P* = 0.01) and ANA positivity, specifically with titers exceeding 320x (HR 4.67, *P* = 0.04), were statistically significant predictors in association with developing APS autoantibodies. Additionally, we conducted Fisher’s exact test to assess the association between TNFi-induced ANA conversion to ≥ 320 × and APS positivity, which observed a trend suggesting a potential correlation between these two conditions (*P* = 0.109).

**Table 5:** Multivariate analyses predicting APS autoantibody positivity.

Predictors	Multivariate	
	HR (95% CI)	*P* value
Female sex	1.42 (0.40–5.07)	.59
Initiation age of biologics	1.00 (0.96–1.04)	.94
BMI	1.05 (0.98–1.13)	.17
PASI	1.11 (1.03–1.20)	.01
Concurrent HT or DL or DM	1.92 (0.52–7.07)	.33
Disease duration prior to the initiation of biologics, years	0.96 (0.91–1.01)	.13
Concurrent arthritis at the initiation of biologics	2.18 (0.67–7.13)	.20
ANA positivity (titers higher than 40x)	0.51 (0.14–1.94)	.33
ANA positivity (titers higher than 320x)	4.67 (1.06–20.6)	.04

*HR* hazard ratio, *CI* confidence interval, *BMI* body mass index, *PASI* Psoriasis Area and Severity Index, *HT* hypertension, *DL* dyslipidemia, *DM* diabetes, *ANA* antinuclear antibody.

## Discussion

Previous studies have reported that ANA antibodies are frequently observed in patients treated with TNFi compared to those treated with IL-17i or IL-23i^7,15–17^. This study aimed to investigate the frequency of APS-associated autoantibodies in patients with psoriasis receiving different biologics treatments, TNF, IL-17 or IL-23 inhibitors. The results revealed that patients treated with TNFi exhibited higher frequencies of ANA and APS autoantibodies compared to those treated with IL-17i or IL-23i. Additionally, within the TNFi-treated group, patients with APS autoantibody positivity showed a higher prevalence of concurrent arthritis and elevated PASI scores at the initiation of treatment. The study also identified ANA titers exceeding 320 × or higher PASI scores as a risk factor for developing APS autoantibody positivity. We were not able to collect pre-treatment data for APS autoantibodies in the present study. This absence restricts our ability to fully understand the baseline status of these antibodies in our patient cohort. Importantly, there are no existing reports suggesting an inherently higher prevalence of APS autoantibodies in psoriasis patients, independent of biologics treatment. In addition, the positive threshold for APS autoantibodies is set above the 99th percentile of healthy individuals^18^, indicating that the prevalence of positivity in the healthy group is nearly zero. This information may support the potential effects of therapeutic agents on APS autoantibody positivity against the baseline with a minimal prevalence of APS autoantibodies.

The autoantibody production during TNFi therapy is well-documented and is not exclusive to APS autoantibodies^19,20^. Although the precise mechanism underlying this process remains elusive, TNFi may involve alterations in immune homeostasis by activating the IFN-γ/Th1 pathway and IFNα, which may potentially contribute to the production of autoantibodies^21,22^. This process underscores the complex interplay within the IFNα-TNFα-IL-23-IL17 pathway core to psoriasis pathology. While TNF inhibitors modulate the pathway in a manner that reduces IL-17 levels, inhibitors directly targeting IL-23 or IL-17 pathways may have a less direct and pronounced effect on IFNα activation compared to TNFi. Moreover, IL-17 itself has been implicated in stimulating autoantibody production, suggesting that IL-17i could potentially suppress autoantibody production more effectively than TNFi^23^. In addition, it has recently been reported that TNFi-stimulated peripheral blood mononuclear cells (PBMCs) may induce prolonged persistence of autoreactive T cells and increase the antigen-presenting capacity of antigen-presenting cells by decreasing the production of IL-2 and IL-10, which may also contribute to autoantibody production^24^. In addition to the altered immunological milieu of TNFi, the effects of TNFi themselves on apoptotic pathways have also been implicated in autoantibody production^25,26^. TNF inhibitors are known to influence apoptotic pathways and may inadvertently impact CD44 expression, a molecule integral to cell adhesion and migration^27,28^. This alteration can lead to an increased production of autoantibodies due to the accumulation of post-apoptotic cellular debris, which may potentially trigger autoantibody production. Despite this well-established association of TNFi and autoantibody production, it is noteworthy that none of the patients in our study exhibited clinical symptoms of APS syndrome. This observation underscores individual differences in immunological tolerance and reactivity. The presence of autoantibodies alone may not precipitate a clinical syndrome, highlighting the complexity of factors that contribute to the development of autoimmune disorders.

In the present study, an increase in the prevalence of concurrent arthritis and higher PASI scores were observed in patients with APS autoantibody positivity, suggesting an intricate association between inflammation and autoimmunity. Topical administration of TLR7 agonists, frequently utilized in animal models of psoriasis, initially triggers Th17-type inflammation; however, prolonged and continuous topical application leads to the production of autoantibodies^29^, wherein systemic inflammation may induce a breakdown of self-tolerance, particularly within the context of innate immune activation^30^. Based on this, the pathogenesis of psoriasis involving the IFNα-TNFα-IL-23-IL-17 pathway suggests that the elevated levels of IFNα in severe psoriasis cases might be further increased by TNF inhibitors, potentially heightening the risk of autoantibody production. This consideration is crucial for understanding the complex interplay between psoriasis severity and autoimmune predisposition. In support of this hypothesis, a substantial number of complications related to autoimmune diseases associated with autoantibody production have been reported in psoriasis cases^31^. In this study, ANA antibody titers exceeding 320 × correlated favorably with APS autoantibody positivity, while no correlation was detected in the group with titers of 40 × or higher in multiple regression analysis (Table 5). Considering that higher titers of ANA are associated with an enhanced capacity of autoantibody production, autoimmune activation may have triggered the production of APS autoantibodies in conjunction with ANA^6^. Thus, patients with ANA titers of 320 × or higher in clinical practice should be recommended to be closely monitored for APS autoantibodies, as higher ANA titers may serve as a sign of altered autoimmune regulation.

The association between the emergence of ANA in psoriasis patients following TNFi treatment and the subsequent loss of treatment response is well-documented^7,14^. In the present study, we aimed to extend this understanding by assessing therapeutic response in APS autoantibody-positive patients, utilizing the frequency of therapy switches as an indicator of treatment effectiveness. The findings showed no significant difference in therapy switching between APS autoantibody-positive and -negative patients, suggesting that APS status may not substantially affect TNFi courses. Our analysis was limited by the lack of evaluation based on PASI scores due to insufficient follow-up data on these scores within our patient cohort, which may induce certain limitations to the interpretation of our findings. Additionally, the potential role of anti-drug antibodies (ADA) was not explored in our study because of the unavailability of stored serum samples for testing. The presence of such antibodies could significantly influence treatment outcomes and efficacy^14^. Future research could significantly benefit from detailed tracking PASI scores and information on ADA presence, allowing for a more comprehensive understanding of treatment responsiveness in APS autoantibody-positive patients.

The present study has several additional limitations. The analysis was performed at a single center, which may limit the generalizability of our findings. Importantly, confirmation of APS autoantibody-associated antibodies ideally requires two measurements at least 12 weeks apart^32^; however, in our cohort, remeasurements were not conducted universally as all patients had no clinical symptoms of thrombosis or embolism. This is a significant limitation, as it impacts the definitive diagnosis of APS autoantibody positivity and the clinical significance of autoantibody positivity is still unknown. A more extended prospective follow-up study is desirable to fully understand the clinical implications of autoantibody positivity and to inform future treatment strategies. In addition, multiple regression analysis identified ANA conversion to ≥ 320 × as a significant predictor of APS positivity, however, additional analysis exploring the correlation between TNFi-induced ANA conversion to ≥ 320 × and APS positivity by Fisher’s exact test did not reach statistical significance (*P* = 0.109). It should be noted that there were missing values in our datasets for both ANA titers with pre- and post-treatment as well as for APS data, which could potentially affect the outcome of our statistical test. Further analysis with a more complete dataset would reveal a possible link between ANA conversion and APS positivity.

In summary, to our knowledge, this is the first study to investigate the association between TNFi and increased frequency of APS-associated autoantibody production. The findings indicate that elevated PASI scores and high ANA titers serve as predictors for susceptibility to APS antibody positivity, underscoring the immunomodulatory interplay between autoimmunity and inflammation in the pathogenesis of psoriasis.

## Data Availability

The datasets generated and analyzed under ethical considerations and privacy restrictions during the current study are not publicly available but are available from the corresponding author on reasonable request.
